# Gastrointestinal Bleeding and Direct Oral Anticoagulants among Patients with Atrial Fibrillation: Risk, Prevention, Management, and Quality of Life

**DOI:** 10.1055/s-0041-1730035

**Published:** 2021-06-16

**Authors:** Paolo Zappulla, Valeria Calvi

**Affiliations:** 1 Division of Cardiology, Centro alte specialità e trapianti (C.A.S.T.), Azienda Ospedaliero-Universitaria Policlinico “G. Rodolico - San Marco,” University of Catania, Catania, Italy

**Keywords:** oral anticoagulant, gastrointestinal bleeding, warfarin, rivaroxaban, dabigatran, edoxaban, apixaban

## Abstract

A significant problem for patients undergoing oral anticoagulation therapy is gastrointestinal bleeding (GIB), a problem that has become increasingly urgent following the introduction of direct oral anticoagulants (DOACs). Furthermore, in recent years a greater focus has been placed on the quality of life (QOL) of patients on long-term oral anticoagulant therapy, which necessitates changes in lifestyle, as well as posing an increased risk of bleeding without producing objective symptomatic relief. Here, we examine current evidence linked to GIB associated with oral anticoagulants, with a focus on randomized control trials, meta-analyses, and postmarketing observational studies. Rivaroxaban and dabigatran (especially the 150-mg bis-in-die dose) appeared to be linked to an increased risk of GIB. The risk of GIB was also greater when edoxaban was used, although this was dependent on the dose. Apixaban did not pose a higher risk of GIB in comparison with warfarin. We provided a summary of current knowledge regarding GIB risk factors for individual anticoagulants, prevention strategies that lower the risk of GIB and management of DOAC therapy after a GIB episode.

## Introduction

Novel direct oral anticoagulants (DOACs) have proven to work effectively in preventing and treating thrombosis. Therefore, they now represent the primary therapeutic drugs in the prevention of both venous thromboembolism (VTE) and ischemic stroke in atrial fibrillation (AF). 1 The guidelines published by the European Society of Cardiology (ESC) regarding the management of AF contain a class-IA recommendation, that, DOACs are recommended as the drug of choice for preventing AF in place of warfarin. 2 In comparison to warfarin, DOACs have shown rapid onset and offset of action, predictable pharmacodynamics thus eliminating the need for regular therapeutic monitoring, and fewer food-drug or drug-drug interactions. 3 While multiple meta-analyses and phase-IV studies have demonstrated that DOACs have a favorable safety profile, high-risk patients are still at risk of bleeding, particularly gastrointestinal bleeding (GIB). Several studies have shed light on how GIB is determined by the DOAC regimen. 4 5 Patient quality of life (QOL) can be affected during treatment with an oral anticoagulant, since it may necessitate lifestyle changes and increase risk of bleeding without providing objective symptom relief. Better treatment adherence is linked to greater satisfaction regarding anticoagulant treatment, and thus to an improved QOL. 6 7 Health-related QOL (HRQOL) is usually assessed by questionnaires that evaluate patients globally, not considering their diagnosis, such as Short Form (SF) 36 or the World Health Organization (WHO) QOL-BREF. 8 9 10 11 12 13 Here, we summarized the evidence currently available with regard to GIB in the context of DOAC treatment used to lower the risk of stroke and systemic embolus in AF. This was performed via a PubMed search using the terms “gastrointestinal bleeding and anticoagulants,” and gathering evidence restricted to the past 7 years, as well as previously published references that were deemed significant. All material was subsequently screened and the reports considered most relevant were selected, including meta-analysis or systematic reviews of randomized clinical trials (RCTs) in first place. After these came, single-clinical trials, meta-analysis of observational studies, or quality single observational studies featured a sample size greater than 100 observations. The main source of information was provided by RCTs of anticoagulation in patients with AF.

## Risk of Gastrointestinal Bleeding with Direct Oral Anticoagulants

### Pharmacology of Direct Oral Anticoagulants

The anticoagulation effects of DOACs are exerted by targeting single enzymes. Apixaban, edoxaban, and rivaroxaban are inhibitors of factor Xa, while thrombin is inhibited directly by dabigatran. These characteristics allow for the administration of a predictable dose without the need for plasma monitoring of coagulation factors. Dabigatran etexilate is a prodrug that, following oral administration, is converted to its active form, where it then functions as a reversible and competitive direct thrombin inhibitor. Multiple doses in healthy volunteers show the drug to have a half-life of 12 to 14 hours, although in patients whose creatinine clearance (CrCl) is less than 30 mL/min, this increases to more than 24 hours. It was noted that 80% of dabigatran is eliminated by renal excretion. It can be taken at a dose of 150-mg bis in die (b.i.d.). However, this should be lowered to 110-mg b.i.d. in cases of renal deficiency, along with CrCl < 50 mL/min.

Dabigatran is instead contraindicated in the presence of severe renal impairment, CrCl < 30 mL/min or in cases of advanced liver disease. 1 14 Rivaroxaban functions as an oxazolidinone-derived anticoagulant that inactivates factor Xa. As a result, the intrinsic and extrinsic pathways of the blood coagulation cascade are interrupted. Following oral administration, plasma concentration reaches its maximum after 1 to 4 hours. While the effects last approximately 12 hours, factor Xa activity requires more than 24 hours to return to normal levels, so it can therefore be taken once a day. Elimination is 50% hepatobiliary and 35% renal, therefore patients with CrCl ≤ 15 mL/min or child class-B or -C liver disease should not be administered rivaroxaban. 1 15 It is given at a dose of 20 mg daily, or 15 mg daily if CrCl is < 50 mL/min. One other oral selective inhibitor of factor Xa is apixaban, 30% of the drug is eliminated via renal excretion with a half-life of approximately 5 hours. The kidneys excrete 25% of the absorbed drug, with a half-life of approximately 12 hour. Apixaban is taken at a dose of 5-mg b.i.d., although this should be lowered to 2.5 mg if patients fall into two or more of the following categories: aged 80 years or over, body weight 60 kg or less, or serum creatinine 1.5 mg/dL or more. 1 5 An oral direct inhibitor of factor Xa that resembles both rivaroxaban and apixaban is edoxaban. It reaches maximum serum concentrations within 1 hour to 2 hours and is characterized by 50% renal excretion of 60 mg daily. However, this goes down to 30 mg daily if the patient presents CrCl < 50 mL/min or weighs less than 60 kg. Edoxaban is contraindicated in patients with severe renal impairment (CrCl < 15 mL/min) and advanced liver disease. 5 16 The characteristics of different DOACs are summarized in Table 1 .

**Table 1:** Characteristics of different novel oral anticoagulants

Characteristics	Dabigatran	Rivaroxaban	Edoxaban	Apixaban
Mechanism of action	Antithrombin	Antifactor Xa	Antifactor Xa	Anti-factor Xa
Bioavailability	3–7%	66% without food, 80–100% with food	50%	62%
Tmax (h)	1.5	2.5	3	1–5
T ½ (h)	12–17	5–9 (young) 11–13 (elderly)	12	10–14
Dosing	b.i.d	Once daily	b.i.d	Once daily
Clearance non renal (%)/renal of absorbed dose (%)	20/80	65/35	73/27	50/50
Liver metabolism: CYp3A4 involved	No	18%	25%	<4%
Absorption with food	No effect	+35% more (therefore needs to be taken with food)	No effect	6–22% more; minimal effect on exposure

Abbreviations: b.i.d., bis in die; Tmax, time to peak plasma level; T ½, half-life.

### Risk of Direct Oral Anticoagulants–Related Gastrointestinal Bleeding in Randomized Clinical Trials

The efficacy and safety of DOACs have been studied in several clinical trials and risk of GIB depends on the DOAC regimen. The four landmark phase-III RCTs are summarized in Table 2 .

**Table 2:** Major DOAC RCTs

Drug and dose compared with warfarin	Clinical trial	Relative risk and 95% IC
Dabigatran 150-mg twice daily	RE-LY	1.48 (1.18–1.85)
Dabigatran 110-mg twice daily	RE-LY	1.08 (0.85–1.38)
Rivaroxaban 20-mg once daily	ROCKET-AF	1.61 (1.30–1.99)
Apixaban 5-mg twice daily	ARISTOTELE	0.89 (0.70–1.15)
Edoxaban 60-mg once daily	ENGAGE-TIMI 48	1.23 (1.02–1,50)
Edoxaban 30-mg once daily	ENGAGE-TIMI 48	0.67 (0.53–0.83)

Abbreviations: CI, confidence interval; DOAC, direct oral anticoagulant; RCT, randomized clinical trial.

RE-LY was an RCT in which warfarin was compared with dabigatran at twice-daily doses of 110 and 150 mg, respectively. 17 In comparison with warfarin and dabigatran administered at a dose of 110 mg, dabigatran 150 mg twice daily was linked to an increased prevalence of GIB (hazard ratio [HR]: 1.48; 95% confidence interval [CI]: 1.18–1.85 and HR: 1.36; 95% CI: 1.09–1.70, respectively). However, incidences of major GIB in patients given twice-daily doses of dabigatran 110 mg did not increase. 18

The ROCKET-AF trial compared patients taking rivaroxaban at a dose of 20 mg daily (reduced to 15 mg in cases of CrCl = 30–50 mL/min) with those taking warfarin. Results indicated a greater prevalence of GIB, both major and minor, in those who were given rivaroxaban (HR: 1.42; 95% CI: 1.22–1.66). 19 A second analysis of the ROCKET-AF trial revealed a higher incidence of major GIB in patients aged 75 years or older: 2.81/100 patient-years versus 1.41 in those aged below 75 years. 20

The ARISTOTLE trial indicated that the rate of major bleeding with apixaban 20 mg was 2.13% per year, as opposed to 3.09% per year in the group administered warfarin group (HR: 0.69; 95% CI: 0.60–0.80). Respective death rates due to any cause were 3.5 and 3.9% (HR: 0.89; 95% CI: 0.80–0.99). The possibility of major GIB associated with apixaban 20 mg was similar to that of warfarin, with advanced age increasing the risk. In patients taking apixaban instead of warfarin, there was less of a risk of nonmajor bleeding including GIB (HR: 0.69; 95% CI: 0.63–0.75). 21

The ENGAGE AF-TIMI 48 trial studied the administration of edoxaban 60 mg daily, edoxaban 30 mg daily, and warfarin. The primary objectives of the study were the prevention of stroke or systemic embolism, wherein neither dose of edoxaban was inferior to warfarin. They were, however, linked to lower incidences of bleeding and death from cardiovascular causes. High doses of edoxaban were linked to a higher annualized rate of major GIB (1.51%) compared with warfarin (1.23%). However, the rate was lowest with low-dose edoxaban (0.82%). 22

The GIB risk of DOACs has been assessed in several systematic reviews and meta-analyses. A meta-analyses of phase-III RCTs showed that, compared with warfarin, rivaroxaban (risk ratio [RR]: 1.46; 95% CI: 1.2–1.8), high dosage of edoxaban (RR: 1.22; 95% CI: 1.01–1.47) and dabigatran (RR: 1.50; 95% CI: 1.20–1.88) significantly increased bleeding while null effect was detected with apixaban. 23 Another meta-analysis pooled the results of 19 RCTs and was thus able to analyze 75,081 patients. It indicated a higher risk of GIB associated with DOACs in comparison with standard care (RR: 1.45; 95% CI: 1.07–1.97). 24 The study was a standard and high-quality meta-analysis, including all available RCTs. However, it was limited in two ways: first, both major and minor GIB were combined in establishing the outcome, and second, acute coronary syndrome studies were included in which controls were placebos and antiplatelet agents provided the basis for the administration of DOACs. Such an approach ran the risk of producing biased results and thus overestimating the risk of major GIB. An opposite result was reported in a 2015 study by Caldeira et al 24 25 which used a precise definition of major GIB and pooled data by all indications. AMPLIFY-EXT and RE-SONATE, two trials that compared the effects of DOACs with placebos, were also included. The authors reported that there was no increased risk of major GIB associated with any of the individual DOACs. It should be noted that different controls (vitamin-K antagonists [VKAs], low molecular weight heparin [LMWH], aspirin, and placebo) were used to obtain the results for each individual DOAC. This approach, however, was restricted by a limited sample size in each subgroup, inevitably lessening the statistical power of the results.

A more recent meta-analysis included a total of 43 randomized trials, totaling 166,289 patients. They showed no difference between DOACs and conventional anticoagulants in the risk of major bleeding (1.5 vs. 1.3%, respectively; HR: 0.98; 95% CI: 0.80–1.21) or clinically relevant nonmajor bleeding (0.6 vs. 0.6%, respectively; HR: 0.93; 95% CI: 0.64–1.36). Dabigatran (2.0 vs. 1.4%, respectively; HR: 1.27; 95% CI: 1.04–1.55) and rivaroxaban (1.7 vs. 1.3%, respectively; HR: 1.40; 95% CI: 1.15–1.70) were linked with an increased likelihood of major GIB when compared with conventional anticoagulation. However, no such difference was observed for apixaban (0.6 vs. 0.7%, respectively; HR: 0.81; 95% CI: 0.64–1.02) or edoxaban (1.9 vs. 1.6%, respectively; HR: 0.93; 95% CI: 0.78–1.11). 26 Therefore, the data indicate that an increased risk of GIB is associated with use of dabigatran and rivaroxaban compared with warfarin. However, it should be noted that the patients who participated in these trials had different numbers of comorbidities and risk factors, thus a cautious approach to this conclusion is advisable.

### Risk of Direct Oral Anticoagulants–Related Gastrointestinal Bleeding in Observational Studies

Since the majority of RCTs adhered to stringent inclusion and exclusion criteria, so as to include only those patients with a relatively low risk of GIB, it may not be possible to generalize the results with respect to the general population. Furthermore, RCTs that separately investigated GIB did so only for major GIB. Thus, the risk of all GIB may have been underestimated. 24 Studies performed by Graham et al 27 and Hernandez et al 28 utilized population-based data which revealed that dabigatran was associated with an increased rate of GIB relative to warfarin in patients with AF.

In a retrospective cohort study conducted by Hernandez et al, 1,302 patients taking dabigatran were compared with 8,102 patients taking warfarin. The study analyzed four subgroups of high-risk patients as follows: (1) those with chronic kidney disease, (2) African American patients, (3) patients aged 75 years or over, and (4) patients with seven or more concomitant comorbidities. Results indicated that all subgroups were subject to a greater risk of major GIB (HR: 1.85; 95% CI: 1.64–2.07), highlighting African American patients and patients with chronic kidney disease as those most at risk. 28

In a study by Abraham et al, 29 rivaroxaban or dabigatran was not found to differ when compared with warfarin, with the exception of patients aged 75 years or over where both drugs were associated with an increased risk of GIB. However, other studies indicated that these drugs did not increase the risk. 30 31 32 In a recent propensity-matched cohort study in patients with AF, results revealed that rivaroxaban, dabigatran, and apixaban were associated with increased, equivalent, and decreased risks, respectively, when compared with warfarin. 33

Several observational studies have compared the risk of GIB with respect to different DOAC regimens. However, the results were limited due to several factors such as inconsistent definition of GIB, selective bias as a result of the observational nature of the study, lack of comparative study of all DOAC regimens, and it are often observed prescription of lower doses of DOACs and poor adherence among patients. Therefore, it becomes difficult to make a comparison between these results and those from meta-analysis.

The current data, when viewed together, indicate possible variability across DOACs with regard to GIB risk. They also highlight increased probability of GIB associated with rivaroxaban and dabigatran. However, such a link was not suggested for apixaban or edoxaban. This potential difference in GIB risk has yet to be explained in terms of biology. With respect to dabigatran, one possible cause could be the tartaric acid coating which exerts a direct caustic effect on the intestinal lumen. DOACs have also been shown to present some degree of intraluminal anticoagulant activity as a result of incomplete absorption across the GI mucosa. This is not the case for warfarin, which is almost completely absorbed and parenteral anticoagulants which are not taken orally. 34 Such a hypothesis could be useful in explaining why dabigatran and rivaroxaban are associated with an increased risk when compared with conventional therapy. However, the difference among DOACs remains unclear. It should also be noted that, independent of GIB risk, all four studied agents were associated with a lower risk of intracranial bleed.

### Risk Factors for Gastrointestinal Bleeding with Anticoagulants

Varying factors have been associated with a higher risk of major GIB in patients administered DOACs. Observational studies and RCTs both commonly investigate risk factors, particularly observational studies, due to the fact that high-risk patients are often left out by RCTs.

Several studies suggest that patients aged 75 years or over were at a greater risk of GIB associated with DOACs. 29 30 35 36 Since elimination of DOACs is dependent on renal excretion, there is a higher likelihood of drug accumulation in patients with impaired renal function, and therefore a higher risk of bleeding. 28 Patients with a past history of peptic ulcer disease or GIB are at a 2.3-fold increased risk of GIB. 30 Another well-known risk factor is concomitant antiplatelet therapy. 30 37 38 Ethnicity was also established as a risk factor. This was observed in Chinese patients administered dabigatran where a higher incidence rate of GIB (4.2 per 100 person-years) was recorded. 39 This is in contrast to the lower incidence rate observed in western populations (1.2–1.5 per 100 person-years in Denmark and 0.6 to 3.4 per 100 person-years in the United States). 34 35 This difference could be explained by genetic factors, in particular, the V Leiden mutation which is extremely rare in Asians. 40 A higher risk of GIB was also associated with liver cirrhosis (HR: 5.6; 95% CI: 1.7–18.8) 41 which increases the likelihood of both gastric or esophageal varices as a complication of portal hypertension and coagulation function abnormalities, and thus GIB.

In a recent study, chronic obstructive pulmonary disease (COPD) was associated with increased risk of GIB (HR: 4; 95% CI: 1.4–11.2), although the precise mechanism behind this has yet to be explained. However, it is plausible that patients with COPD may have a long history of smoking which is a known risk factor for acute GIB. 36 41 Analysis of data from 114,835 patients indicated that a higher risk of GIB was associated with concomitant use of oral anticoagulants (OACs) and other gastrotoxic drugs when compared with any of the drugs used alone: OAC + nonsteroidal anti-inflammatory drugs (NSAIDs; RR: 8.7; 95% CI: 7.3– 10.4), OAC + aspirin (RR: 6.9; 95% CI: 5.9–8.2), and OAC + COX-2 inhibitors (coxibs; RR: 5.0; 95% CI: 3.2–7.8). 42 When OACs are used concurrently with other drugs, such as gemfibrozil, a higher risk of GIB was also observed (HR: 1.8; 95% CI: 1.4–2.4). 43 Anemia is often seen in patients with AF, and it could possibly be linked to a greater risk of new-onset AF. 44 45 VKAs also put patients with anemia at a greater risk of bleeding. 46 47 48 However, majority of randomized controlled trials of DOACs have not included patients with hemoglobin <10 g/dL. 17 21 24 49 Furthermore, the current guidelines contain no specific recommendation regarding anticoagulant therapy in anemic patients with AF and hemoglobin <10 g/dL in current guidelines. 2 50 A recent cohort study classified 8,356 patients with AF into two subgroups as follows: (1) patients with hemoglobin ≥10 g/dL and (2) patients with hemoglobin <10 g/dL. Compared with warfarin, DOACs were associated with a reduced risk of major bleeding or gastrointestinal tract bleeding in patients with <10 g/dL (HR: 0.43; 95% CI: 0.30–0.62). However, no such difference was observed in the incidence of ischemic stroke, systemic embolism, or death in anemic (HR: 0.79; 95% CI: 0.53–1.17). 51 Heart failure (HF) and AF often coexist. 52 Patients with HF are at a higher risk of bleeding in comparison to non-HF controls. 53 A history of HF also serves as a greater predictor of major bleeding than of thromboembolic risk. 54 The efficacy and safety outcomes of DOACs compared with warfarin in patients with AF and HF have been examined by several studies. A meta-analysis conducted recently observed no difference in HF and GIB (RR: 1.11; 95% CI: 0.79–1.55). 55

### Prevention of Gastrointestinal Bleeding Associated to Direct Oral Anticoagulants Treatment

Before DOACs are prescribed, a strategy should be implemented to minimize risk. Should this fail, the identified risk factors should be considered so as to choose an appropriate prescription and dose.

Initially, the bleeding risk in patients with AF taking warfarin was derived by using the HAS-BLED (hypertension, abnormal liver/renal function, history of stroke, bleeding tendency, labile international normalized ratios [INRs], elderly aged ≥65 years, and drug/alcohol use; Table 3 ) score. 56 57 A score of ≥3 indicates a high-risk patient, with a score of 3, conferring 3.74 bleeding events per 100 patient years. It is important to recognize, however, that patients with a greater risk of thromboembolism usually present one or more of the comorbidities listed in the HAS-BLED criteria. They are therefore more predisposed to bleeding. DOACs should not be prescribed until patients have been screened for hepatic and kidney disease. In this way, a drug can be chosen which is compatible with the patient's comorbidities ( Tables 4 and 5 ). Drug interactions must be considered, as patients are commonly prescribed multiple drugs. This is especially important with drugs that are metabolized via the cytochrome P450 system and the P-gp efflux transporter. If possible, clarithromycin, fluconazole, itraconazole, amiodarone, cimetidine, rifampicin, carbamazepine, phenobarbital, and protease inhibitors should be not be used. 58 This is also the case with combinations of any anticoagulant used with NSAIDs and coxibs. However, cases may arise where this is not possible. Should this occur, coxibs are preferred to traditional NSAIDs. 59 A history of peptic ulcer may necessitate testing for *Helicobacter pylori* to prevent upper GIB. Proton pump inhibitor (PPI) treatment lowers the probability of upper GIB, 60 61 62 but it is possible that the preventive effect of taking DOACs is not as great as that observed when PPIs are given in conjunction with gastrotoxic drugs such as NSAIDs or aspirin. 63 64 It has been suggested that PPIs potentially interact with anticoagulants due to their shared liver metabolism via P450-cytocrome. However, this was ruled out in a multicenter case-control study. 65 PPIs will not, however, prevent GIB from the lower GI tract, and further investigation is warranted regarding the potentially negative effect they have on intestinal microbiota. 66

**Table 3:** Items of HAS-BLED 56 57 bleeding risk score

Clinical characteristics	Definition	Points
Hypertension	Systolic blood pressure > 160 mm Hg	1
Abnormal liver or renal function	Chronic liver disease (e.g., cirrhosis) or biochemical evidence of significantly impaired liver function (e.g., bilirubin > 2 times the ULN plus one or more liver enzymes > 3 times the ULN Chronic dialysis, renal transplantation, or serum creatinine ≥ 200 µmol/L	1 or 2
Stroke	Previous history of stroke	1
Bleeding tendency or predisposition	Bleeding disorder or previous bleeding episode requiring hospitalization or transfusion	1
Labile INRs	Labile INRs in patients taking warfarin (failure to maintain a therapeutic range at least 60% of the time)	1
Elderly	Age > 65 years	1
Drugs	Concomitant antiplatelet agents or NSAIDs excessive alcohol use (≥ 8 units per week)	1 or 2

Abbreviations: INR, international normalized ratio; NSAID, nonsteroidal anti-inflammatory drugs; ULN, upper limit of normal.

**Table 4:** Use of DOACs according to renal function

CrCl	Dabigatran	Rivaroxaban	Edoxaban	Apixaban
≥50 mL/min	2 × 150 mg	20 mg	60 mg	2 × 5 mg or 2 × 2.5 mg a
50–30 mL/min	2 × 150 mg or 2 × 110 mg b	15 mg	30 mg	2 × 5 mg or 2 × 2.5 mg a
30–15 mL/min	No	15 mg	30 mg	2 × 2.5 mg
Dialysis	No	No	No	No

Abbreviations: CrCl, creatinine clearance; DOAC, direct oral anticoagulant.

Note: 2 × 2.5 mg only if at least two out of the following three: age ≥ 80 years, body weight ≤ 60 kg, and/or creatinine ≥ 1.5 mg/dL.

a 2 × 110 mg in patients at high risk of bleeding.

b Other dose reduction criteria may apply (weight ≤ 60 kg, concomitant potent P-Gb inhibitor therapy).

**Table 5:** Use of DOACs in liver failure

Child–Pugh category	Dabigatran	Rivaroxaban	Edoxaban	Apixaban
A	No dose reduction	No dose reduction	No dose reduction	No dose reduction
B	Use with caution	Do not use	Use with caution	Use with caution
C	Do not use	Do not use	Do not use	Do not use

Abbreviation: DOAC, direct oral anticoagulant

### Location of Gastrointestinal Bleeding: Upper versus Lower

The lower GI tract is a common source of GIB in DOAC users. 67 In a post hoc analysis of patients experiencing GIB during RELY, 47% of patients taking dabigatran 110-mg twice daily and 47% of patients taking dabigatran 150-mg twice daily were noted to have experienced lower GI bleeding. In contrast, only 25% of warfarin-associated GIB was from the lower tract. 35 Dabigatran appears to increase the risk of lower GIB compared with warfarin. A retrospective study found a 30% increased risk of lower GIB with dabigatran (HR: 1.30; 95% CI:1.04–1.62). 68 In the ROCKET-AF trial, patients taking rivaroxaban had similar rates of upper and lower GIB as patients on warfarin: 48 and 47% of rivaroxaban and warfarin users, respectively, had upper GIB; 22 and 24% had lower GIB, respectively; and 30 and 29% had rectal bleeding, respectively. 4 Similarly, in a postmarket retrospective cohort study of GIB in anticoagulated patients, 57% of patients taking rivaroxaban were found to have a lower GI source, as were 75% of the patients taking dabigatran. 69 . In ARISTOTLE, the most frequent specified site of major bleeding in patients taking apixaban was the digestive tract. Apixaban showed similar rates in terms of the location of bleeding; the event rate for upper versus lower GI bleeds in patients taking apixaban was 0.43 per 100 patient-years versus 0.25 per 100 patient-years. The event rate for upper versus lower GI bleeds in patients taking warfarin was 0.56 per 100 patient-years versus 0.24 per 100 patient-years. 37 In the ENGAGE-AF-TIMI trial, the annualized rate of major bleeding events was 3.43% with warfarin versus 2.75% with high-dose edoxaban and 1.61% with low-dose edoxaban. The corresponding rates for upper GIB were 0.71, 0.91, and 0.56 with warfarin, high-dose edoxaban, and low-dose edoxaban, respectively; these rates were 30 to 50% higher than those reported for lower GIB (0.52, 0.62, and 0.28, respectively). 22

## Management of Direct Oral Anticoagulants Therapy after a Gastrointestinal Bleeding Episode

Patients taking DOACs who present with overt, nonmajor GIB require specific management consisting of drug cessation and endoscopic management. 34 70 71 Should a non–life-threatening major bleeding event occur in patients with normal renal function, plasma levels of DOACs should normalize within 12 to 24 hours, although patients with renal insufficiency may require more time, particularly for dabigatran. 34 50 In cases of severe bleeding and/or hemodynamic instability, options may include activated charcoal, hemodialysis/hemoperfusion, and reversing anticoagulation.

**Fig. 1:**
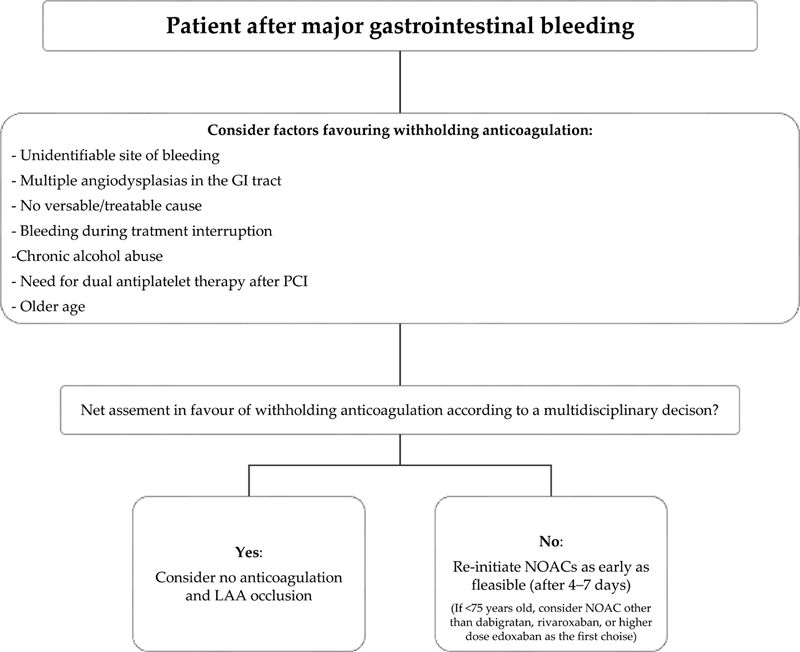
Management of anticoagulant therapy after major gastrointestinal bleeding. GI, gastrointestinal; LAA, left atrial appendage; NOAC, non–vitamin K antagonist oral anticoagulants; PCI, percutaneous coronary intervention.

If the last dose of DOAC is given within 2 hours, activated charcoal can be used to reduce intestinal absorption of residual drug. This potential benefit must be weighed against subsequent impairment of endoscopic visualization. 72 Should life-threatening GIB or renal failure occur, hemodialysis or hemoperfusion could also be considered for dabigatran. 73 However, they should not be used for direct factor Xa inhibitors due to the fact they are highly protein bound. 71 Nonspecific reversal agents include prothrombin complex concentrates (PCCs; either weight based three-factor or four-factor PCCs), activated (aPCCs) and recombinant factor VIIa (rFVIIa), although they have been shown to increase the risk of thromboembolism. 74 75 However, a recent study has demonstrated that four-factor PCCs possess a similar safety profile when compared with fresh frozen plasma in terms of thromboembolic events (around 7%) and deaths. 75 Due to their uncertain efficacy and potential risk of thromboembolism, these agents should only be administered in the following situations: life-threatening GIB, ongoing bleeding despite standard measures, or delayed clearance of DOACs in patients with renal failure. 70 Availability of PCC and aPCC, as well as the experience of the treatment center, generally determine which is used. This is particularly true in the case of aPCC which, since it induces a strong procoagulant effect, should only be administered by physicians with prior experience in its use. PCC and aPCC are preferred over rFVIIa due to the absence of outcome data and the latter's strong procoagulant effect. 76 77 Antifibrinolytic agents (tranexamic acid) have been used to manage DOAC-related GIB, particularly in cases of severe bleeding where many factors of the coagulation cascade are often deficient, but the experience is still limited. 70 78 Specific reversal agents have also been developed. Idarucizumab is a humanized monoclonal antibody fragment (Fab) against dabigatran which has been shown to be capable of rapidly reversing the anticoagulation activity of dabigatran within minutes in almost all patients. 79 Andexanet alfa is a recombinant modified human factor Xa decoy protein which functions as a universal factor Xa reversal agent by binding to the factor Xa inhibitors. 70 It has been shown to greatly reverse antifactor Xa activity and promote hemostasis in approximately 80% of patients with acute major bleeding. 79 The severity of GIB and patients' hemodynamic status determines the timing of endoscopy. In situations of mild GIB, it is possible to defer endoscopic evaluation for 12 to 24 hours. 31 75 80 This delayed approach presents many advantages, such as increasing effectiveness of endoscopic intervention once the drug effects have worn off, increasing safety in a nonemergency setting, and improving endoscopic visualization due to attenuation/cessation of bleeding and better colonic cleansing. However, patients who either have GIB or hemodynamically unstable should undergo emergency endoscopy promptly after resuscitation. If endoscopic management fails, radiological and/or surgical interventions should be considered as a last resort. 81 It is possible to restart nuisance or minor bleeding anticoagulation in the majority of cases, sometimes done by delaying or skipping a single dose. In other cases of bleeding, particularly those which are life-threatening, careful reassessment of the risks and benefits or restarting anticoagulation is required. Specific data regarding restarting DOAC after GIB is lacking, thus there is an absence of randomized data on restarting medication post-GIB. The benefits and risks of resuming anticoagulant therapy following GIB were examined in a meta-analysis performed by Chai-Adisaksopha et al 82 which involved the selection of three studies, including patients on warfarin for various reasons. Where warfarin was resumed (in 53% of patients), a substantial reduction in thromboembolic events (9.9 vs. 16.4%, HR: 0.68; 95% CI: 0.52–0.88) and mortality (24.6 vs. 39.2%, HR: 0.76; 95% CI: 0.66–0.88) was observed. However, it also revealed a numerically increased rate of recurrent GIB in cases where warfarin was resumed (10.1 vs. 5.5%, HR: 1.20; 95% CI: 0.97–1.48; *p* = 0.10). The risk greatly increased where warfarin was resumed within 7 days as opposed to resuming later. In accordance with these findings, the European Society of Gastrointestinal Endoscopy (ESGE) guideline recommends that patients taking DOAC with moderate-to-severe GIB cease DOAC and resume 7 to 15 days after the GIB episode. Patients at an increased thrombotic risk, such as those with mechanical heart valve, cardiac assist device, or CHA2 DS2-VASc score ≥4 may benefit from earlier (first week) resumption. 83 However, many additional factors should also be considered. This is especially true in situations of severe and life-threatening bleeding without an obvious secondary or reversible/treatable cause, where the potential benefits may not be worth the risks of resuming anticoagulation. Should such a case arise, implantation of a left atrial appendage (LAA) occluder, or surgical LAA occlusion may represent a potential substitute for long-term anticoagulation. LAA occlusion appears to be a promising option for AF patients who are not candidates for long-term OAC. Recently increasing evidence of the utility of LAA occlusion in patients who are not candidates for long-term oral anticoagulation. Long-term data from two continued access registries, PROTECT-AF 84 and PREVAIL, 85 to support LAA occlusion as a safe and effective long-term anticoagulation therapy. This new evidence led to class-IIb recommendation for left atrial appendage occlusion (LAAO) in nonvalvular atrial fibrillation patients not eligible for long-term OAC. PRAGUE-17 randomized controlled trial showed LAA occlusion is noninferior to DOAC. 86 Additionally, a lower dose or apixaban should be considered for patients with GIB while on DOAC 2 ( Fig. 1 ).

## Outcomes of major gastrointestinal bleeding (MGIB)

Several studies have indicated that patient outcomes in cases of major bleeding into any organ while taking DOACs are no worse and might perhaps be more favorable than during VKA treatment. 66 In RE-LY, the rate of life-threatening GIB was similar in patients treated with dabigatran 110-mg b.i.d. and warfarin. It increased, however, where dabigatran 150-mg b.i.d. was used ( *p* < 0.004). 87 Furthermore, patients on dabigatran who were experiencing major bleeding required fewer plasma transfusions but more packed red blood cell transfusions. They also spent less time in intensive care units and benefitted from an decreased mortality rate when compared with patients taking warfarin. 88 Among patients with major GIB, ROCKET-AF found similar incidences of life-threatening bleeding, death, and transfusion of greater than 4 units of packed red blood cells in those taking rivaroxaban and warfarin. 4 The ENGAGE-AF and ARISTOTELE studies also associated major GIB and DOACs with favorable outcomes in patients administered edoxaban and apixaban when compared with VKA. 26 89 Data from the Dresden registry would also seem to support favorable outcomes for users of DOAC with major GIB, with a favorable rate of DOAC continuation compared with those taking VKA. 90

## Patients' Quality of Life

The extremely variable biological effects provoked by warfarin and their narrow therapeutic index make it a complicated drug to use. 91 92 Patients who have undergone treatment with warfarin must therefore been seen more frequently at outpatient clinics to monitor their INR. Dietary restrictions must also be put in place to reduce vitamin-K intake, since it can adversely affect patients' HRQOL). 93 While clinical studies have mainly attempted to evaluate the efficacy and safety of anticoagulant therapy, they have not focused so much on HRQOL. Period blood monitoring is not necessary to assess the efficacy of DOACs. They are also characterized by a wide therapeutic window and low inter- and intraindividual variability in dose-effect relation with fewer interactions between drugs. 94 Several studies have examined how HRQOL is affected by OAC therapy. 95 Corbi et al 96 in their study recorded worse scores of HRQOL among women, the elderly, patients with less than 1 year of therapy, and those with an indication other than metallic prosthetic heart valve for OAC use. Warfarin was the most prescribed OAC in their study group, at 83.1%. In another study conducted by Lancaster et al, 97 no substantial difference between patients on warfarin and the control group were observed in terms of HRQOL until a bleeding episode had occurred. Their conclusion stated episodes of bleeding, such as GIB, led to patients experiencing a significant decrease in perceived health. A recent study indicated that DAOCs have comparable QOL, greater treatment satisfaction, reduced hospitalization, and a nonsignificant trend toward fewer bleeding episodes when compared with warfarin. 89 Major GIB events in DOAC users are generally associated with favorable outcomes which is an important aspect that can influence HRQOL. 90 In a European survey discontinuation related to bleeding was evident in only 4% of the patients. 98 The preferences of AF patients toward anticoagulation shows that stroke risk reduction and limited bleeding risk are the most important attributes for an AF patient when deciding whether they are for or against a certain treatment. 99

The Hospital Anxiety and Depression Scale (HADS) represents a reliable and valid tool in the assessment of depression and anxiety in patients and the general population. 100 A recent study revealed a positive correlation between HADS scores and the annual number of hospital admissions, indicating that the warfarin group was more likely to suffer from depression and anxiety. When patients with effective INR levels were analyzed, HADS scores were seen to increase among those with ineffective INR levels. This can most likely be explained by the difficulties associated with gaining an effective INR level which have a detrimental effect on patients' emotional wellbeing and thus increase HADS scores. 101 In conclusion, it is possible that DOACs improve symptoms of anxiety and depression in nonvalvular AF patients which would thus lead to a better HRQOL assessment and lower HADS scores in the DOAC group.

## Conclusion

This article summarizes the current literature about GIB in patients on anticoagulant therapy. However, there is a lack of direct comparisons between DOACs. Current understanding is thus based on an unfortunately limited amount of evidence taken from observational studies and indirect comparisons in meta-analyses of RCTs. Rivaroxaban and dabigatran (particularly the 150-mg twice daily dose) are seemingly associated with a greater risk of GIB. This risk is also increased when edoxaban is administered, although it is dependent on the dose. With apixaban, the risk of GIB does not appear to increase in comparison to warfarin. Therefore, it is of the utmost importance that DOAC indications be reviewed and that a particular DOAC be prescribed on an individual basis. Physicians must also be aware of the risk factors for DOAC-related GIB and adopt the necessary preventive measures. Furthermore, higher levels of HRQOL were recorded among patients treated with DOACs as opposed to those treated with warfarin. These results may be linked to a lower rate of GIB events and fewer patients requiring hospitalization.
